# Chromosome X Open Reading Frame 38 (CXorf38) Is a Tumor Suppressor and Potential Prognostic Biomarker in Lung Adenocarcinoma: The First Characterization

**DOI:** 10.3390/proteomes13020022

**Published:** 2025-06-03

**Authors:** Rui Yan, Heng-Wee Tan, Na-Li Cai, Le Yu, Yan Gao, Yan-Ming Xu, Andy T. Y. Lau

**Affiliations:** 1 The Second Affiliated Hospital of Shantou University Medical College, Shantou 515041, China; 18ryan@stu.edu.cn (R.Y.); hwtan@stu.edu.cn (H.-W.T.); 24ygao@stu.edu.cn (Y.G.); 2 Laboratory of Cancer Biology and Epigenetics, Department of Cell Biology and Genetics, Shantou University Medical College, Shantou 515041, China; nlcai@stu.edu.cn (N.-L.C.); 22lyu1@stu.edu.cn (L.Y.); 3 The First Affiliated Hospital of Shantou University Medical College, Shantou 515041, China

**Keywords:** CXorf38, lung cancer, ORF protein, uncharacterized protein

## Abstract

**Background:** Previously, we found that an uncharacterized protein CXorf38 is significantly downregulated in human ZIP8-knockout (KO) cells. Given that ZIP8 regulates essential micronutrients linked to diseases including cancer, this study aims to characterize CXorf38 and evaluate its role in lung adenocarcinoma. **Methods:** iTRAQ-based proteomics was previously used to identify the abundance of proteins in ZIP8-KO cells. Cell proliferation and colony formation assays were used to examine the function of CXorf38 by overexpressing the gene in lung adenocarcinoma cell lines. Kaplan–Meier survival analysis was used to assess the prognostic value of *CXorf38*, while TCGA clinical database analysis was used to evaluate its expression in lung cancer tissues, particularly in smokers. Bioinformatics analyses (GO, KEGG, PPI, and ICI) were performed on *CXorf38*-coexpressed genes derived from patients with lung cancer. **Results:** CXorf38 overexpression suppressed lung cancer cell proliferation and colony formation, suggesting a tumor-suppressive role. Higher *CXorf38* expression correlated with improved survival in patients with lung adenocarcinoma, but not in lung squamous cell carcinoma. Clinical data showed *CXorf38* downregulation with lung cancer tissues of smokers, indicating a potential role in smoking-induced cancer progression and treatment. Functional analysis using bioinformatics linked CXorf38 to immune response regulation, suggesting involvement in the tumor immune microenvironment. **Conclusions:** Our study reveals for the first time that CXorf38 is a potential tumor suppressor, prognostic biomarker, and/or tumor immune regulator in lung adenocarcinoma—further research is warranted to explore its role in tumor immunity and its therapeutic potential.

## 1. Introduction

Proteins are fundamental biomolecules that play crucial roles in virtually all biological processes, including but not limited to structural support, enzymatic reactions, signaling, and immune responses [1]. Compared to DNA and RNA, proteins exhibit greater complexity and diversity due to their intricate structures, dynamic interaction networks, and post-translational modifications (PTMs) [2].

Owing to the rapid advancement of proteomics technology, we can now efficiently detect the status of biomolecules of interest (e.g., abundance of proteoforms including isoforms, PTMs in proteins, and protein–protein interaction [PPI]) on a large scale and in high-throughput settings [1,3,4]. Despite the vast amount of proteomics data generated over the years, many proteins remain uncharacterized—these “uncharacterized proteins” are commonly referred to as open reading frame (ORF) proteins [5,6]. Recent studies on certain ORF proteins have revealed their diverse biological functions [7], and these ORF proteins have functions involving cell development [8], DNA damage repair [9], gene expression [10], and so on. However, the potential involvement of ORF proteins, including their proteoforms, in human diseases remains largely unexplored. Therefore, it is crucial to study the functions of ORF proteins in order to better understand the pathophysiology of diseases, including cancer progression.

Previously, we established a ZIP8-knockout (KO) cell model by knocking out the *SLC39A8* gene in the HeLa human cervical cancer cell line using the CRISPR/Cas9 system [11]. The *SLC39A8* gene encodes ZIP8, a transmembrane protein responsible for transporting divalent metal ions into cells [12], and impaired ZIP8 protein has been linked to disease-associated disruptions in cellular ion homeostasis [11,13,14]. To further investigate the biophysiological role of ZIP8, we performed isobaric tags for relative and absolute quantitation (iTRAQ)-based proteomics analysis to assess proteomic changes between the ZIP8-wildtype and ZIP8-KO cells [15]. Among the most significantly low-abundance proteins in the ZIP8-KO cells, we identified an uncharacterized protein: chromosome X Open Reading Frame 38 (CXorf38). In this study, we aim to elucidate the biological function of CXorf38 and investigate its potential role in lung cancer.

## 2. Materials and Methods

### 2.1. Cell Culture

All human lung adenocarcinoma cell lines used in this study were purchased from the American Type Culture Collection (ATCC) (Rockville, MD, USA) and cultured in conditions as recommended by the ATCC. Specifically, Ham’s F-12K complete growth medium was used for the A549 cell line, and RPMI-1640 complete growth medium was used for H1299 and H358 cell lines. Both F-12K and RPMI-1640 medium contained 10% fetal bovine serum, 100 U/mL penicillin, and 100 µg/mL streptomycin. All cell lines were cultured at 37 °C in an atmosphere of 5% CO_2_/95% air as recommended by the ATCC.

### 2.2. Plasmids and Transfection

The plasmids (pEGFP-C1 and pEGFP-N1) used in this study were purchased from Clontech (Mountain View, CA, USA). Plasmids pEGFP-C1-CXorf38 and pEGFP-N1-CXorf38 were constructed in our laboratory (Figure S1). Specifically, the DNA coding region was cloned into pEGFP-C1 or pEGFP-N1 plasmid at the *Hin*dIII and *Bam*HI restriction sites to generate an N- or C-terminal GFP tag, respectively. For pEGFP-C1-CXorf38, the DNA sequence was amplified by PCR using primers (5′-CCCAAGCTTAAGTGCTGTCGGAGCTAGCG-3′ and 5′-CGGGATCCTCAGGCCTTCCTGT CAGGTGTTT-3′) following conditions: initial denaturation at 95 °C for 3 min followed by 25 cycles of denaturation at 95 °C for 30 s, annealing at 62 °C for 60 s, extension at 72 °C for 30 s, and final extension at 72 °C for 7 min. For pEGFP-N1-CXorf38, the DNA sequence was amplified by PCR using primers (5′-CCAAGCTTGCCACCATGGTGCTGTCG-3′ and 5′-CGGGATCCGAGGCCTTCCTGTCAGGTGTTT-3′) following conditions: initial denaturation at 95 °C for 3 min followed by 25 cycles of denaturation at 95 °C for 30 s, annealing at 55 °C for 60 s, extension at 72 °C for 30 s, and final extension at 72 °C for 7 min. Next, PCR products were gel-purified and digested with *Hin*dIII and *Bam*HI restriction enzymes. Then, the purified PCR products were ligated to the vectors using T4 ligase and transformed into *E. coli* strain DH5α for verification by DNA sequencing.

To overexpress CXorf38, A549, H1299, and H358 cells were seeded in a 6 cm dish at 1 × 10^6^, 0.9 × 10^6^, and 0.8 × 10^6^ cells, respectively. After 24 h of culturing, the cells were transfected with pEGFP-N1-CXorf38 or pEGFP-C1-CXorf38 plasmid (6 μg per reaction) using Lipofectamine 2000 reagent (Invitrogen, Carlsbad, CA, USA) in accordance with the manufacturer’s instructions.

### 2.3. Immunoblotting Analysis

The expression level of protein was determined using an immunoblotting analysis. To extract proteins, cells transfected with specific plasmids were washed with pre-chilled PBS and collected by scraping. Then, cell pellets were collected via centrifugation (1000× *g* for 5 min at 4 °C) and resuspended by RIPA lysis buffer (50 mM Tris [pH 7.5], 150 mM NaCl, 1% Triton X-100, 0.1% SDS, 1% sodium deoxycholate, 5 mM EDTA, 1 mM NaF, 1 mM Na_3_VO_4_, and protease inhibitor cocktail [Roche Diagnostics GmbH, Penzberg, Germany]). The lysed cell samples were then subjected to sonication (3 times for 6 s) and centrifugation at 12,000× *g* for 10 min at 4 °C. The protein concentrations were measured using the Bradford reagent (Bio-Rad Laboratories, Hercules, CA, USA).

An equal amount of protein lysate for each sample (40 µg) was loaded into and resolved by 10% SDS-PAGE and transferred onto a polyvinylidene difluoride membrane (0.45 µm). The membrane was then blocked in Tris-buffered saline containing 0.1% Tween and 5% non-fat dry milk for 1–2 h at room temperature. Then, the membrane was probed with CXorf38 (PA5-62139; Invitrogen) or β-actin (A5441; Sigma-Aldrich, St. Louis, MO, USA) primary antibodies at 4 °C overnight, followed by incubations with horseradish peroxidase-conjugated anti-rabbit IgG (dilution 1:10,000, 7074S; Cell Signaling Technology, Danvers, MA, USA) or anti-mouse IgG (dilution 1:10,000, AS003; ABclonal, Woburn, MA, USA) secondary antibodies for at least 1 h. Then, enhanced chemiluminescence immunoblotting detection reagent (GE Healthcare, Boston, MA, USA) was used to detect the expression of targeted proteins as described previously in [16], using a Tanon-5200 chemiluminescence image analysis system (Shanghai Tanon Life Science, Shanghai, China).

### 2.4. Cell Proliferation Assay

The effects of CXorf38 on the proliferation of A549 cells were determined by the naphthol blue black (NBB) staining assay, as described previously in [17]. Briefly, after transfection with pEGFP-N1-CXorf38, A549 cells were trypsinized and seeded in 96-well plates at 2000–2500 cells/well in F-12K medium. The cells were then incubated for 12, 24, 36, 48, 60, and 72 h prior to the NBB assay, and the absorbance of the cell suspension was measured at 595 nm using a 96-well multiscanner (Thermo Scientific Multiskan FC, Thermo Scientific, Waltham, MA, USA). An impedance- and microsensor electrode-based Real-Time Cell Analysis (RTCA) technique was also used to quantify cell proliferation using xCELLigence RTCA S16 (ACEA Biosciences, San Diego, CA, USA). Briefly, A549, H1299, and H358 cells were seeded in the 16-well electronic microtiter plates at 5000, 4000, and 5000 cells/well, respectively. The device was placed in a culture chamber (maintained at 37 °C in an atmosphere of 5% CO_2_/95% air) and operated according to the manufacturer’s instructions. Cell growth was detected in a 30 min interval for at least 100 h.

### 2.5. Focus Formation Assay

The effects of CXorf38 on the focus (colony) formation ability of A549 cells were determined by the focus formation assay. Briefly, after transfection with pEGFP-N1 or pEGFP-N1-CXorf38 plasmid, cells were trypsinized and seeded in 6-well plates at 500 cells per well in F-12K medium and cultured for a total of 15 days. Cell foci were stained with crystal violet, and images were captured using a digital scanner (V370 Photo, EPSON, Long Beach, CA, USA). The stained foci were dissolved in 10% acetic acid, and the absorbance was measured at 595 nm.

### 2.6. Kaplan–Meier (KM) Survival Analysis and Detection of CXorf38 Level in Smokers

Survival analysis was performed using the KM plotter database (http://kmplot.com, accessed on 1 March 2025) to assess the correlation between the *CXorf38* level and the survival of patients with lung cancer. Briefly, two datasets of lung cancer were used: the microarray and RNA-seq datasets. The follow-up threshold was set at 120 months. The level of *CXorf38* and smoking status in patients with lung adenocarcinoma were obtained from The Cancer Genome Atlas (TCGA) via cBioPortal for the Cancer Genomics database (www.cbioportal.org, accessed on 1 March 2025). The “Firehose Legacy” dataset was used for the analysis, and the z-score threshold was set at ±1.5.

### 2.7. Identification of CXorf38-Coexpressed Genes, Enrichment Analysis, and PPI

Coexpressed genes of *CXorf38* in patients with lung adenocarcinoma were identified using the same dataset as mentioned above (Firehose Legacy). Briefly, the top 500 most positively or negatively correlated coexpressed genes of *CXorf38* based on Spearman’s correlation value were arranged according to their *p*-value. A positive Spearman’s correlation value (>0 to 1) refers to a positive correlation, while a negative value (<0 to −1) refers to a negative correlation, and *p* ≤ 0.05 is considered statistically significant. These 500 significantly coexpressed genes of *CXorf38* were then subjected to a functional classification of Gene Ontology (GO), using the “ClusterProfiler” R package [18], and the *p*-value was adjusted by Benjamini and Hochberg’s false discovery rate correction. A Kyoto Encyclopedia of Genes and Genomes (KEGG) pathway enrichment analysis and PPI network construction of intersected targets were performed using the Metascape (https://metascape.org, accessed on 5 March 2025) [19].

### 2.8. Immune Cell Infiltration (ICI) Analysis

The immune infiltration and survival prognosis of CXorf38 in lung adenocarcinoma were analyzed using the Tumor Immune Estimation Resource 2.0 (TIMER2.0) database (https://compbio.cn/timer2, accessed on 5 March 2025). Advanced algorithms such as TIMER and CIBERSORT were applied to rigorously evaluate the levels of ICI within lung adenocarcinoma based on TCGA data, and representative results were selected and displayed. The correlations between *CXorf38* and CD4^+^/CD8^+^ T cell infiltration levels were explored by Spearman’s correlation test. A KM overall survival curve for lung adenocarcinoma stratified by CD4^+^ or CD8^+^ T cell levels and *CXorf38* expression was created.

### 2.9. Statistical Analysis

Statistical analysis was performed using GraphPad Prism^®^ 8 software (v8.0.2, GraphPad Software Inc., Boston, MA, USA). Error bars represent the mean ± standard deviation from at least three biological replicates. The two-tailed Student’s *t*-test was used to determine significant differences between the means of two selected groups unless mentioned otherwise. A *p* ≤ 0.05 was considered statistically significant.

## 3. Results and Discussion

### 3.1. Proteomics Reveals Significant Downregulation of CXorf38 in ZIP8-KO Cells

**Figure 1:**
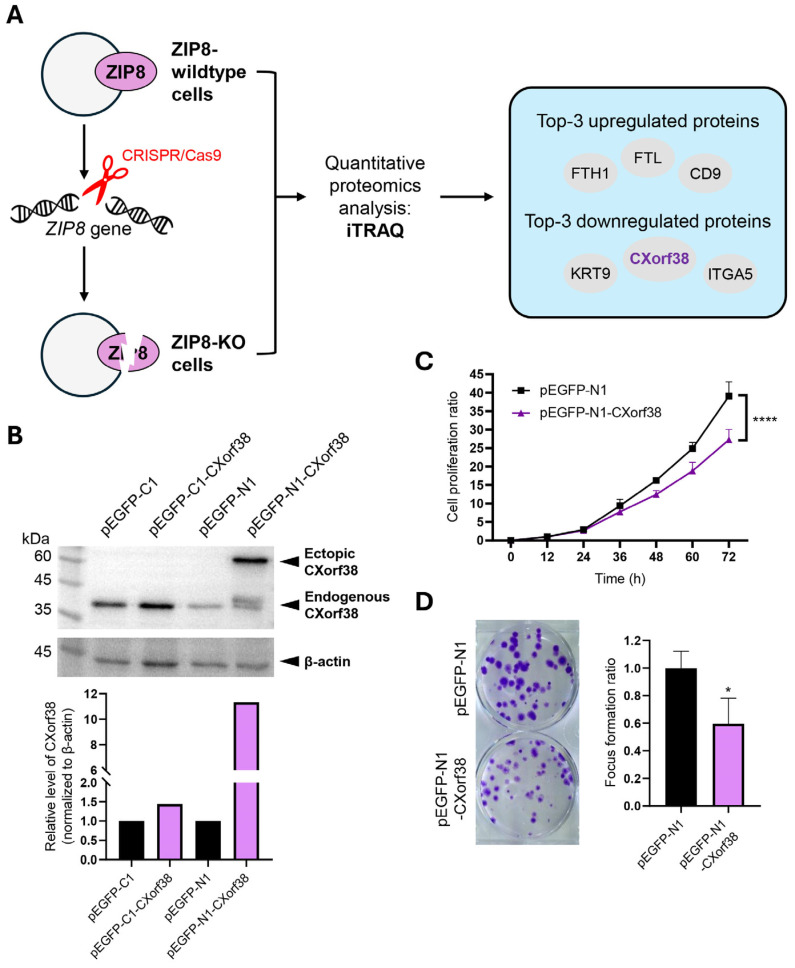
(**A**) Schematic representation of the experimental protocol as described previously in [15]. Briefly, a ZIP8-KO cell model was established by knocking out the *ZIP8* gene in the genome of the HeLa cell line using the CRISPR/Cas9 system. Both ZIP8-wildtype and ZIP8-KO cell lines were then subjected to isobaric tags for relative and absolute quantitation (iTRAQ)-based proteomics analysis, and the abundances of proteins were identified. (**B**) Overexpression of CXorf38 in A549 cells verified by immunoblot analysis using antibodies against CXorf38 (PA5-62139; Invitrogen) and β-actin (A5441; Sigma-Aldrich) (see Figure S2 for full blots). The quantification of the immunoblot result was performed using Gel-Pro^®^ Analyzer 4.0. (**C**) Cell proliferation of CXorf38-overexpressed and control cells was analyzed using NBB assay. (**D**) Focus formation assay of CXorf38-overexpressed and control cells cultured for 15 days. * *p* < 0.05; **** *p* < 0.0001.

Previously, we detected the proteomes of a ZIP8-KO cell line using iTRAQ and LC-MS/MS techniques, and we identified CXorf38 as one of the lowest-abundance proteins [15] (Figure 1A).

ZIP8 is a transmembrane protein responsible for transporting Zn as well as other essential micronutrients [12,20]. Studies have shown that impaired ZIP8 function can disrupt intracellular levels of key micronutrients such as Fe, Mn, Se, and Zn [15,21], which may contribute to a range of human diseases, including cancer [21,22,23].

Although the direct connection between ZIP8 and CXorf38 is unclear, we previously demonstrated that the CXorf38 level was decreased in ZIP8-KO cells and could be induced by selected essential micronutrients [15]. Specifically, the protein expression of CXorf38 significantly increased upon exposure to essential micronutrients Mn and Se, but there were no significant changes when exposed to Zn [15]. These results suggest that CXorf38 expression is regulated by cellular micronutrient levels, and CXorf38 may be involved in the downstream pathways of nutrient homeostasis, transporter regulation, metal response, and cellular stress response. However, despite these findings, the biological function of CXorf38 remains largely unknown.

### 3.2. CXorf38 Suppresses Growth and Colony Formation in Lung Cancer Cells

In this study, we sought to better elucidate the biological function of CXorf38 by investigating its role in lung cancer. We first overexpressed CXorf38 in lung adenocarcinoma cell line A549 using two plasmids (pEGFP-C1 and pEGFP-N1), and immunoblot analysis data showed that only the cells transfected with pEGFP-N1-CXorf38 successfully overexpressed CXorf38 (Figure 1B). Thus, these cells, along with pEGFP-N1 control-transfected cells, were selected for subsequent experiments.

Through cell proliferation and focus formation assays, we found that the growth and colony formation ability of CXorf38-overexpressed A549 cells were being suppressed (Figure 1C,D). To further verify these data, we used the RTCA method to detect the cell proliferation rates of the A549 cell line and two other lung adenocarcinoma cell lines (H1299 and H358) transfected with pEGFP-N1 or pEGFP-N1-CXorf38, and the results indicated that all three CXorf38-overexpressed lung cancer cell lines proliferated slower than their control counterpart (Figure S3). Since cell proliferation rate and colony formation ability are common phenotypes of cancer cells that could dictate cancer progression [24,25], the above results suggest that CXorf38 may play an anti-cancer role in lung adenocarcinoma. However, it remains unclear whether CXorf38’s role is specific to lung adenocarcinoma or conserved across different cancer types. Also, it is worth noting that multiple bands are observed in the immunoblot probed with CXorf38 antibody in all the CXorf38-overexpressed cell lines (Figures S2 and S3), suggesting that proteoforms could arise from the CXorf38-EGFP fusion protein. However, the conditions for these bands to appear remain unexplored, and further proteoform analyses of CXorf38 are needed in the future. To further explore the potential role of CXorf38 in lung cancer, we conducted subsequent bioinformatics analyses.

### 3.3. CXorf38 as a Prognostic Biomarker for Lung Adenocarcinoma but Not for Lung Squamous Cell Carcinoma

**Figure 2:**
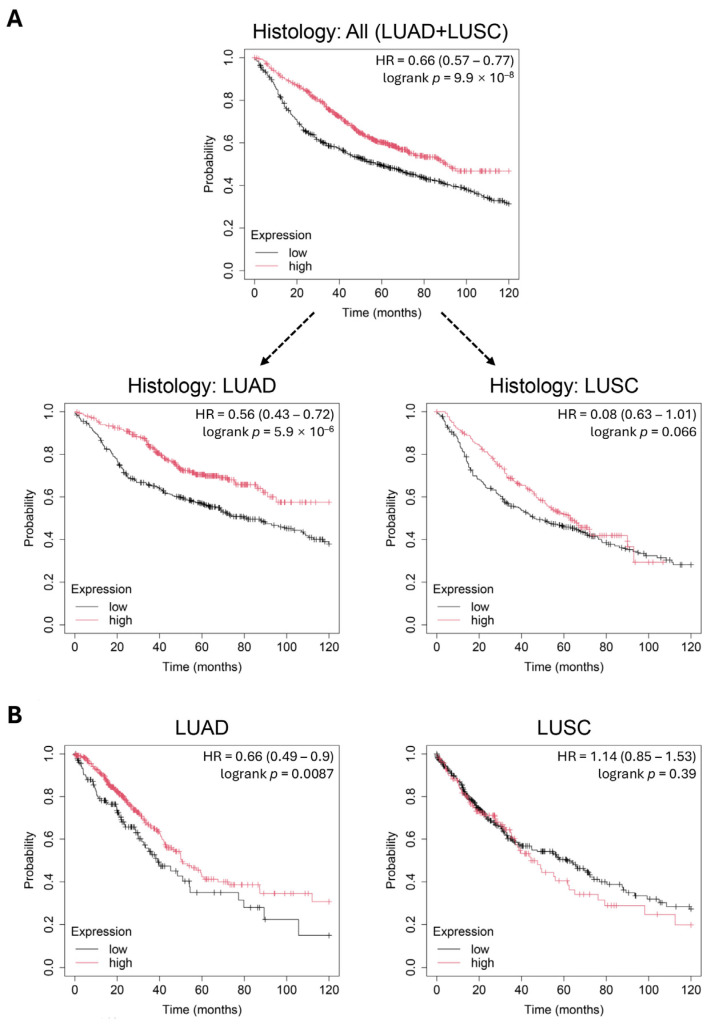
The Kaplan–Meier plotter database was utilized to study the correlation between *CXorf38* expression and the survival of patients with lung cancer. Overall survival analysis was conducted based on microarray (**A**) and RNA-seq (**B**) datasets in the Kaplan–Meier plotter database. The follow-up threshold was set at 120 months. LUAD: lung adenocarcinoma; LUSC: lung squamous cell carcinoma.

Next, we analyzed the prognostic significance of CXorf38 in lung cancer using two independent datasets (microarray and RNA-seq) from the KM plotter database [26]. Our analysis of the microarray dataset indicated that higher *CXorf38* expression correlates positively with survival in patients with lung cancer (Figure 2A). However, when stratified by cancer histology, lung adenocarcinoma showed statistically significant results but not for lung squamous cell carcinoma (even though the *p*-value was very close to 0.05) (Figure 2A). Similar results were obtained using the RNA-seq dataset (Figure 2B). Collectively, these findings indicate that CXorf38 is a prognostic biomarker for lung adenocarcinoma but not lung squamous cell carcinoma.

Similarly to our current findings, *CXorf38* and 11 other genes were previously identified as some of the most significant survival-associated genes in urothelial cancer [27]. Among these genes, high *CXorf38* expression was correlated with favorable survival outcomes, but whether it could regulate the malignancy of cancer cells in urothelial cancer remains to be further studied.

### 3.4. Clinical Database Analysis: CXorf38 Is Downregulated in Lung Cancer Tissue of Smokers

**Figure 3:**
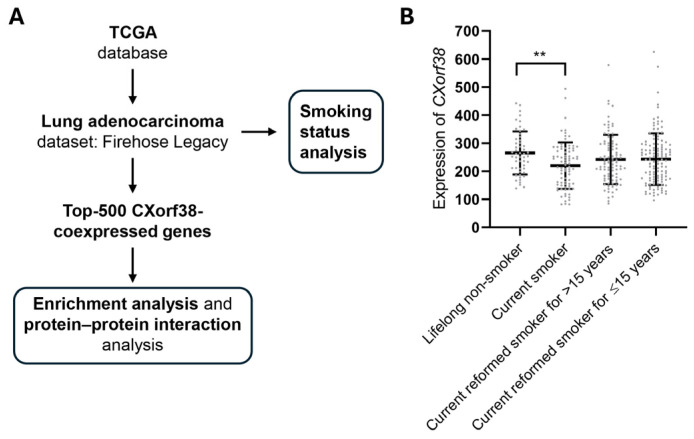
(**A**) Schematic representation of the experimental design used to study the roles of CXorf38 in lung cancer using clinical database analysis. (**B**) Levels of *CXorf38* in lung adenocarcinoma samples (*n* = 348) derived from TCGA database. Error bars represent the mean ± standard deviation. The two-tailed Student’s *t*-test was used to determine significant differences between the means of two groups. ** *p* < 0.01.

We then further investigate the potential roles of CXorf38 in lung cancer via clinical database analysis using the data obtained from TCGA (Figure 3A). We noticed that the CXorf38 protein and gene levels were generally not significantly altered between lung tumor samples and paired normal tissues (31 upregulated and 24 downregulated samples in a total of 517 samples). Interestingly, when we looked at the samples based on patients’ smoking history, we found that *CXorf38* was significantly downregulated in smokers compared to lifelong non-smokers and current reformed smokers (Figure 3B).

It is well established that cigarette smoking promotes cancers, since cigarettes contain multiple carcinogens, including cadmium [28,29]. Epidemiological studies have indicated a strong correlation between smoking and lung cancer [30], and findings in our study (Figure 1C and Figure 3B) revealed that smoking could suppress the level of *CXorf38* in lung tissue, which might contribute to the promotion of lung cancer growth or progression.

### 3.5. Bioinformatics Analysis Unveils the Potential Roles of CXorf38 in the Tumor Immune Microenvironment

To further explore the potential biological function of CXorf38, we identified the genes that were most correlated with the *CXorf38* level in lung cancer samples and performed GO and KEGG pathway enrichment analyses with the top 500 most positively or negatively correlated coexpressed genes of *CXorf38*.

**Figure 4:**
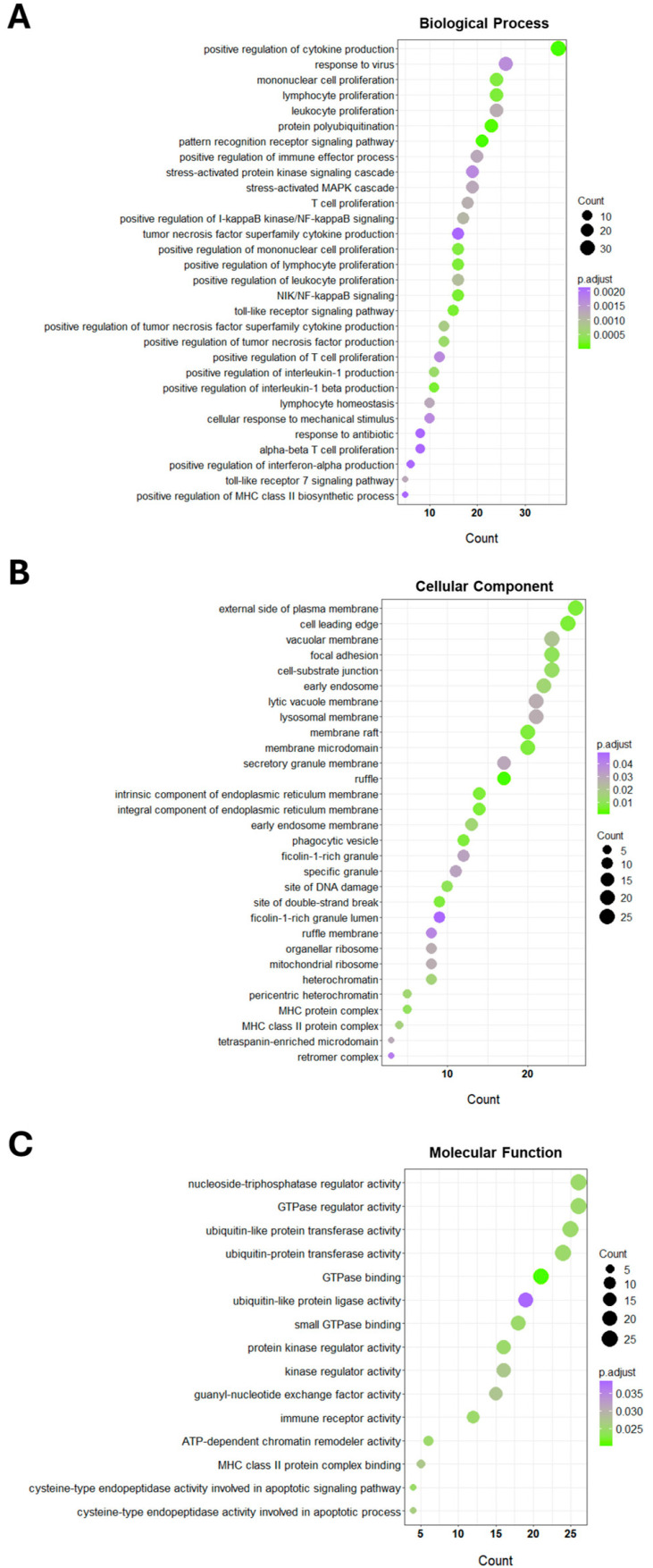
Bubble plot of Gene Ontology (GO) enrichment analysis of the top 500 *CXorf38*-coexpressed genes in lung cancer samples described in Figure 3A. Selected genes were classified using the GO categories: (**A**) biological process, (**B**) cellular component, and (**C**) molecular function.

Briefly, results from the GO enrichment analysis indicated that the most important biological processes of these *CXorf38*-related genes were mainly associated with cellular immune responses (Figure 4A; Table S1), revealing the potential roles of CXorf38 in the tumor immune microenvironment. For GO cellular component enrichment analysis, the top five enriched outcomes were “external side of plasma membrane”, “cell leading edge”, “vacuolar membrane”, “focal adhesion”, and “cell-substrate junction” (Figure 4B; Table S2). For molecular function enrichment analysis, it was shown that many genes were associated with ubiquitination and GTPase (Figure 4C; Table S3). Notably, pathways such as “immune receptor activity” were also among the most significantly enriched GO molecular functions (Figure 4C).

**Figure 5:**
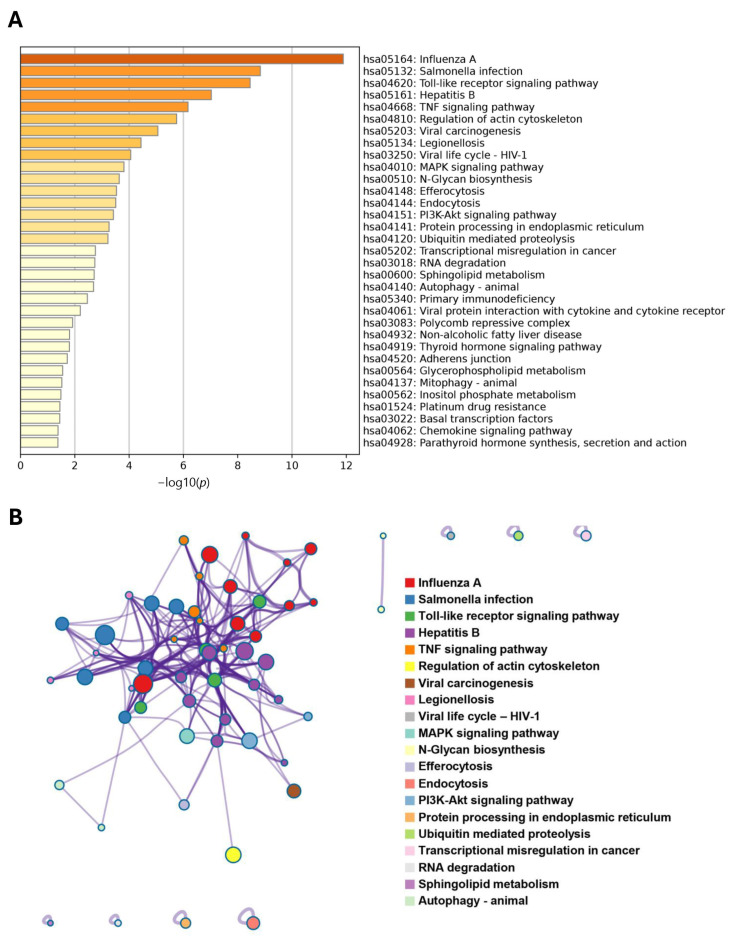
(**A**) Kyoto Encyclopedia of Genes and Genomes (KEGG) enrichment analysis and (**B**) protein–protein interaction (PPI) network of KEGG-enriched terms of the top 500 *CXorf38*-coexpressed genes in lung cancer samples described in Figure 3A.

Similarly, in the KEGG enrichment analysis, several pathways related to immune responses and viral infections were enriched (Figure 5A). Furthermore, pathways that could contribute to cancer progression were also identified—for example, “Toll-like receptor signaling pathway”, “MAPK signaling pathway”, “N-glycan biosynthesis”, and “platinum drug resistance”—and the interactions between these pathways of KEGG are further analyzed and mapped in Figure 5B.

**Figure 6:**
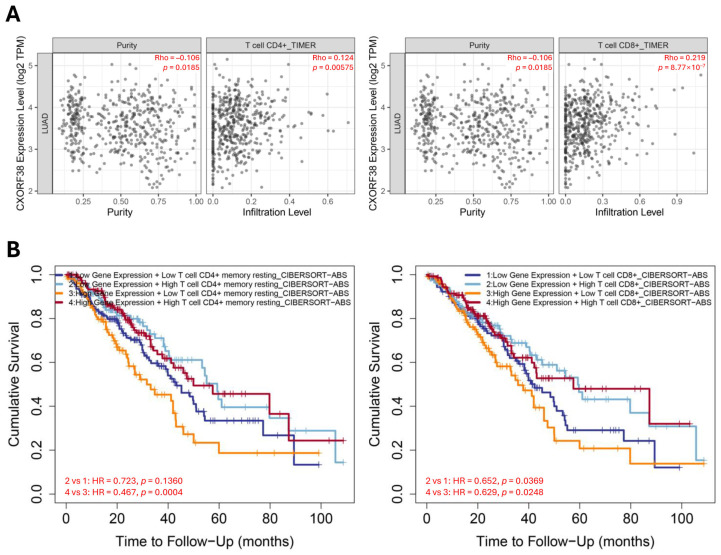
(**A**) Correlation between *CXorf38* and CD4^+^/CD8^+^ T cell infiltration level in lung adenocarcinoma based on TIMER algorithm (TIMER2.0 database). (**B**) KM overall survival curve for lung adenocarcinoma stratified by CD4^+^ or CD8^+^ T cell levels and *CXorf38* expression based on CIBERSORT algorithm.

The tumor microenvironment and ICI are two important regulators of tumor growth and progression [31,32]. To further verify the relationship between CXorf38 and immune infiltration, we conducted a correlation analysis using the TIMER2.0 database. The results revealed a significant positive correlation between *CXorf38* expression and ICI in lung adenocarcinoma (Figure 6A), in which this correlation was also linked to improved overall survival in patients with lung adenocarcinoma with high expression of *CXorf38* stratified by CD4^+^ T cell level (Figure 6B). However, when stratified by CD8^+^ T cell level, it was shown that a higher level of CD8^+^ T cells was always beneficial for survival regardless of *CXorf38* expression level (Figure 6B). Nevertheless, the results obtained in our study suggested that CXorf38 might be involved in regulating the immune microenvironment of tumors, which could have implications for the survival of patients with lung cancer. Overall, further studies are warranted to validate the above bioinformatic data as well as to elucidate the underlying mechanisms of CXorf38 in lung cancer.

### 3.6. Limitations

It is important to acknowledge several limitations of the current study. First, our findings are primarily based on a cell culture model supported with bioinformatics analyses, which provide valuable insights into CXorf38 but require further experimental validation (e.g., in vivo and clinical studies). Second, the mechanism by which CXorf38 inhibits lung cancer cell growth remains unclear, and its underlying molecular mechanism in regulating lung cancer development should be further elucidated. Third, clinical samples are needed to validate the role of CXorf38 in smokers. Fourth, whether the tumor-suppressive role of CXorf38 is specific to lung cancer or conserved across other cancer types remains unclear. Lastly, the proteoforms of CXorf38, which arise due to alternative splicing, post-translational modifications, and potential isoform-specific interactions [33,34,35], have not been explored. These proteoforms of CXorf38 (e.g., multiple bands detected in our immunoblot analysis) may significantly impact its function in lung cancer, making it difficult to pinpoint its precise role in tumor progression or suppression. Despite these limitations, our study highlights the clinical significance of CXorf38, as our findings strongly suggest its potential as a tumor suppressor, prognostic biomarker, and/or immune regulator in lung adenocarcinoma.

## 4. Conclusions

To conclude, our study provides the first functional characterization of CXorf38 in lung adenocarcinoma, highlighting its potential as a tumor suppressor and prognostic biomarker. Its downregulation in smokers suggests a role in smoking-related lung cancer progression and cancer treatment. Furthermore, bioinformatics analysis indicates that CXorf38 could be involved in tumor immune regulation.

## Data Availability

The iTRAQ-based proteomics data used to identify the CXorf38 protein have been deposited in the ProteomeXchange Consortium with the dataset identifier PXD036680 (10.6019/PXD036680) as described previously in [15].

## Supplementary Materials

The following supporting information can be downloaded at https://www.mdpi.com/article/10.3390/proteomes13020022/s1, Figure S1: Plasmid maps of pEGFP-N1-CXorf38 and pEGFP-C1-CXorf38 created using SnapGene^®^; Figure S2: Immunoblot of protein extracted from A549 cells transfected with pEGFP-C1, pEGFP-C1-CXorf38, pEGFP-N1, and pEGFP-N1-CXorf38 plasmids and probed with CXorf38 (PA5-62139; Invitrogen) and β-actin (A5441; Sigma-Aldrich) antibodies. The marker used was a 3-Color Prestained Protein Standards (Accurate Biology, Changsha, Hunan, China); Figure S3: Protein expression levels of CXorf38 in A549 (**A**), H1299 (**B**), and H358 (**C**) cells were verified by immunoblot analysis, respectively, and their cell proliferation rates were assessed by RTCA; Table S1: Data of GO: biological process enrichment analysis of *CXorf38*-coexpressed genes in lung adenocarcinoma; Table S2: Data of GO: cellular component enrichment analysis of *CXorf38*-coexpressed genes in lung adenocarcinoma; Table S3: Data of GO: molecular function enrichment analysis of *CXorf38*-coexpressed genes in lung adenocarcinoma.

## Abbreviations

PTMs	Post-Translational Modifications
PPI	Protein–Protein Interaction
ORF	Open Reading Frame
KO	Knockout
iTRAQ	Isobaric Tags for Relative and Absolute Quantitation
CXorf38	Chromosome X Open Reading Frame 38
ATCC	American Type Culture Collection
NBB	Naphthol Blue Black
RTCA	Real-Time Cell Analysis
TCGA	The Cancer Genome Atlas
GO	Gene Ontology
KEGG	Kyoto Encyclopedia of Genes and Genomes
ICI	Immune Cell Infiltration
